# Calcium carbide-ripened plantain induced alterations in plasma electrolytes concentration and kidney function in rats

**DOI:** 10.1590/2175-8239-JBN-2022-0101en

**Published:** 2023-01-13

**Authors:** Osezele C. Ugbeni, Chidube A. Alagbaoso

**Affiliations:** 1 University of Benin, Faculty of Life Sciences, Department of Biochemistry, Benin City, Nigeria.

**Keywords:** Artificial ripening, Calcium carbide, Plantain, Ripening, Kidney function, Amadurecimento artificial, Carbureto de cálcio, Plátano, Maturação, Função renal

## Abstract

**Introduction:**

Artificial fruit ripening agents such as ethanol, ethylene, ethephon, and calcium carbide (CaC_2_) is usually employed in stimulating the fruit ripening process. Currently, there is a paucity of information regarding the effects of various artificial fruits ripening methods on the health status of consumers. In this study, the physiological effects and possible health hazards associated with the consumption of plantain ripened by CaC_2_ and other non-chemical methods on the kidneys were investigated.

**Methods:**

Artificially ripened plantain was mixed with rat feed and fed to Wistar albino rats for four weeks, and the levels of plasma electrolytes (Na^+^_,_ HCO_3_^−^, K^+^, and Cl^−^), urea, creatinine, as well as histological changes in the kidneys were determined.

**Results:**

Results indicated that rats fed with carbide-ripened plantain had a significantly high level of plasma bicarbonate (HCO_3_^−^) compared to control rats., but there was no difference in the level of plasma sodium (Na^+^). However, the levels of plasma potassium (K^+^) and chloride (Cl^−^) were significantly low in rats fed with CaC_2_-ripened plantain as compared to the control rats. Furthermore, the levels of urea and creatinine were significantly high in rats fed with CaC_2_-ripened plantain compared to the control animals. Histological analyses showed glomeruli atrophy and tubular necrosis in kidneys of rats fed with CaC_2_-ripened plantain, thereby further indicating toxicity to the kidneys.

**Conclusions:**

Histological evidence and alterations in the level of the plasma electrolytes, urea, and creatinine suggest that consumption of fruits ripened with calcium carbide may be harmful to the kidneys.

## Resumo

**Introdução:**

Agentes de maturação artificial como etanol, etileno, etefon e carbureto de cálcio (CaC_2_) são comumente empregados para estimular o amadurecimento de frutas. Atualmente, há uma escassez de informações a respeito dos efeitos de diversos métodos artificiais de maturação de frutas no estado de saúde dos consumidores. Neste estudo, investigaram-se os efeitos fisiológicos e possíveis riscos à saúde associados ao consumo de plátano maturado por CaC2 e outros métodos não químicos nos rins.

**Métodos:**

O plátano artificialmente amadurecido foi misturado com ração de rato e fornecido a ratos albinos Wistar por quatro semanas, e determinaram-se os níveis de eletrólitos plasmáticos (Na^+^, HCO_3_^−^, K^+^, e Cl^−^), ureia, creatinina, bem como alterações histológicas nos rins.

**Resultados:**

Ratos alimentados com plátano amadurecido com carbureto apresentaram um nível significativamente maior de bicarbonato plasmático (HCO_3_^−^) em comparação com ratos controle, mas não houve diferença no nível plasmático de sódio (Na^+^). Entretanto, os níveis plasmáticos de potássio (K^+^) e cloreto (Cl^−^) foram significativamente baixos em ratos alimentados com plátano maturado com CaC_2_ comparados com ratos controle. Além disso, os níveis de ureia e creatinina foram significativamente mais elevados em ratos alimentados com plátano amadurecido com CaC_2_, em comparação com os animais controle. Análises histológicas mostraram atrofia glomerular e necrose tubular em rins de ratos alimentados com plátano amadurecido com CaC_2_, indicando assim ainda mais toxicidade aos rins.

**Conclusões:**

Evidências histológicas e alterações nos eletrólitos plasmáticos, ureia e creatinina sugerem que o consumo de frutas amadurecidas com carbureto de cálcio pode ser prejudicial aos rins.

## Introduction

The natural process of fruit ripening involves a combination of physiological, biochemical and molecular processes^1–3^. Fruit ripening involves coordination of different metabolism with activation and deactivation of various genes leading to change in color, increase in sugar content, decrease in acidity, softening of fruit and increase in flavor and aroma^2, 3, 4, 5^. These processes make fruits to be colored, soft, edible, nutritious, and palatable^1^.

Artificial ripening is done to achieve faster and uniform ripening. It is the process by which ripening is controlled and products may be achieved as per requirement by controlling different parameters^6^. Artificially ripened fruits may develop uniform and attractive surface color but the tissue inside remains green and the fruit generally have shorter shelf life^7^. Various artificial methods of fruit ripening have been observed mostly to meet consumers’ demand and other economic factors. Fruit sellers artificially ripen green fruits even during the due season to meet the high demand and make high profits of seasonal fruits^8^. Transporting and distributing fruits from the farmers’ orchards to consumers’ baskets can take several days. During this time the naturally ripened fruits may become over ripen and inedible. A part of naturally ripened fruits can also be damaged during harsh condition of transportation. It indeed increases great economic loss for the fruit sellers and therefore, to minimize the loss, fruit sellers sometimes prefer collecting fruits before full maturity and artificially ripen fruits before selling to the consumers^9^. Since plantain is a climacteric fruit, it is usually harvested at the pre-climacteric stage and artificially ripened for commercial purposes. Artificial ripening enables traders to minimize losses during transportations as well to timely release the product at desired ripening stage. Plantains can be artificially ripened by different ripening agents^10^.

With the advancement of science and technology, various methods have been developed to artificially stimulate the ripening process, mostly to meet the high demand by consumers. Artificial ripening agents such as ethanol, methanol, methyl jasmonate, ethylene glycol, ethephon, and CaC_2_ are used to ripen fruits and vegetables^11,12^. The plantain plant is an important tropical and subtropical plant, and it is a climacteric plant that belongs to the *Musaceae* family, genus *Musa* and specie *paradisiaca* ^13^. Plantain is estimated to provide about 60 million people in Africa with more than 200 calories per day^14^. The many forms in which they are consumed also indicate the long association between man and the crop^15^. Being a common stable food, plantain is subjected to artificial ripening with different common ripening agents to meet its demand by the large population of consumers.

Plantain is a commonly consumed food in many parts of the world. It supplies necessary calories and essential micronutrients. It is highly perishable, having short shelf life leading to high post-harvest losses of about 20–50% due to poor handling and quality deterioration^16,17^. In order to reduce the high post-harvest losses, plantain is harvested when green but mature, and artificially ripened when needed with the use of ripening agents. Ripening agents are substances that hasten the ripening process, and they come in different forms. Some examples include ethylene gas, ethephon, ethylene glycol, and calcium carbide^18^. The use of artificial ripening agents may give more acceptable color than naturally ripened fruits^19^.

In recent years, artificial fruit ripening has been considered a matter of concern because of various health-related issues^12,20^. CaC_2_ is commonly used to ripen fruits especially in developing countries like Nigeria. The reason for its use is simply because ethylene is produced when water is added to it. Ethylene is a phytohormone that stimulates and regulates the ripening process in plants, and so, calcium carbide being an ethylene producing agent in the presence of water, is usually added to fruits and sprinkled with water to produce ethylene to forcefully stimulate ripening^21^. Several cases of stomach disorder after eating carbide-ripened mangoes have also been reported^12^. Commercial CaC_2_ is known to be contaminated with substantial level of toxic chemicals such as arsenic and phosphorous hydride^22^. Therefore, adding CaC_2_ to fruits in order to stimulate the ripening process may be a dangerous practice as it may affect the health of consumers. Thus, humans are at risk of short term and long-term health effects simply by eating fruits that are artificially ripened. As the demand for fruits as dietary source of minerals, vitamins, and dietary fiber increases, in order to meet the demand, the use of artificial methods to ripen fruits has also increased significantly regardless of the possible health hazards that may result from such methods.

However, the effects of these artificial ripening agents on the nutritional values of fruits, possible toxicity and associated health hazard caused by consuming artificially ripened fruits are yet to be fully understood^9^. This study therefore was aimed at developing a scientific understanding of the changes or effects of the consumption of artificially ripened plantain on kidney functions using animal models.

## Materials and Methods

### Plantain and Ripening Methods

The plantain used for this study were obtained from the University of Benin, Nigeria. The plantain was identified and authenticated by a botanist at the Department of Plant Biology and Biotechnology, University of Benin, and its scientific name was given as *Musa paradisiaca*. The plantain was divided into four groups; The ones that ripened on the tree (on-the-tree ripening), those that ripened on the floor after harvesting (on-the-floor ripening), those that were induced with CaC_2_ (carbide ripening), and lastly those that were ripened using polythene bag (polythene bag ripening). For on-the-tree ripening, the plantain was left on the tree to ripen on its own via the normal ripening process for about 2 weeks and afterwards harvested. For carbide ripening, the plantain was placed inside an empty carton and 218 g of CaC_2_ was sprinkled on them followed by little sprinkle of water, and the carton was properly covered and placed in a dark cupboard. For polythene ripening, the plantain was placed in a polythene bag and placed in a dark cupboard until it was ripe, while for on-the-floor ripening, the plantain was placed on the floor and allowed to ripe on its own without an inducer.

### Rat Diet Preparation

After three days of storage, the naturally ripened plantain had all ripened, but it took five days for the polythene and CaC_2_-induced plantain ripening to take place. Before pulverization, all the plantain peels were removed and discarded, and the weight of the peeled plantain samples was recorded. The plantain was then oven-dried and pulverized. The pulverized plantain was used to formulate diet the animals. The animal feed was composed of 500 g of powdery plantain and 500 g of normal rat feed, which was made into a paste with 50 mL of distilled water, and thereafter made into pellets. The pellets were dried for several days until they hardened up and then used to fed the animals.

### Animals and Dieting

Female Wistar albino rats were purchased and used for this study. The rats were divided into five groups of three animals each. The animals were allowed to acclimatize for two weeks with free access to water and food *ad libitum*. The animals were kept in clean cages, and housed in a well-ventilated room with standard living conditions with their food and water changed every day. The experimental design/animal grouping and diets are shown in Table 1.

**Table 1.** Animal grouping and dieting

Animal groups	Diets
I	Normal Rat feed (control)
II	CaC_2_-ripened Plantain Feed (CPF)
III	Polythene Bag-ripened Plantain Feed (PBPF)
IV	On-The-Floor-ripened Plantain Feed (OFPF)
V	On-The-Tree-ripened Plantain Feed (OTPF)

The animals were fed on these diets for four (4) weeks. After four weeks of feeding, the animals were anaesthetized with chloroform and sacrificed. Blood samples were collected and tissues were harvested for downstream processing and analysis. Plasma electrolytes concentration, (Na^+^, HCO_3_^−^, K^+^, and Cl^–^), urea, and creatinine levels were determined using standard diagnostic kits and following manufacturer's protocols. This study was carried out according to the standard procedure of the University of Benin Animal Use, Welfare, and Regulation.

### Histological Analysis

A section of the rat kidneys measuring about 3–5 mm in thickness was cut out from a kidney tissue preserved in 10% formalin, and placed in a tissue cassette. Leica TP2010 automatic tissue processor was used in processing the tissue section for 18 h, and afterwards fixed in 10% formalin, dehydrated with increasing concentration of isopropyl alcohol, cleared with xylene, and then impregnated with paraffin wax. The tissues were embedded in paraffin wax with an automated tissue embedding machine and cut into ultra-thin sections of five (5) microns. The sections were placed on pre-labelled slides, dried over-night, and subjected to hematoxylin and eosin staining. The stained slides were viewed under a microscope with ×10 and ×40 magnification.

### Data Analysis

All data are expressed as mean ± SEM (n = 3). Statistical significance was determined using one-way ANOVA and GraphPad prism 8 followed by Tukey Kramer tests. P < 0.05 was taken as statistical significance.

## Results

### CaC_2_-Ripened Plantain Altered Plasma Electrolytes Concentration

**Figure 1.**
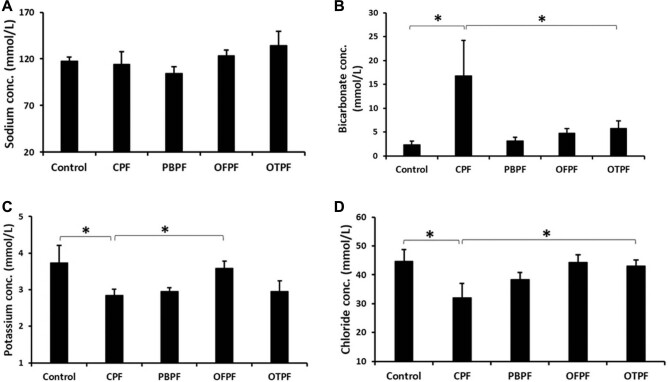
Alterations in plasma electrolytes concentration induced by CaC_2_-ripened plantain. (A) Sodium ion concentration in the plasma of rats. (B) Bicarbonate ion concentration in the plasma of rats that received CaC2-ripened plantain is significantly high. (C) Plasma potassium ion concentration of rats fed with the various diet formulations. (D) Plasma chloride ion concentration of rats fed with CaC_2_-ripened plantain is low. CPF: Carbide-ripened plantain feed, PBPF: Polythene bag ripened plantain feed, OFPF: On-the-floor ripened plantain feed, OTPF: On-the-tree ripened plantain feed. All data are expressed as mean ± SEM (n = 3). *p < 0.05.

Artificially ripened plantains were fed to rats for four (4) weeks and the effect on plasma electrolytes (Na^+^, HCO_3_^−^, K^+^, and Cl^–^) concentration was determined. The results in Figure 1A indicate that the various methods of ripening plantain did not result in alteration of sodium ion concentration. However, plasma bicarbonate concentration was significantly increased in rats fed with CaC_2_-ripened plantain compared to rats in other groups (Figure 1B). Furthermore, a significant decrease in potassium and chloride concentrations were recorded in rats fed with CaC_2_-ripened plantain when compared to the control group and rats that received plantain ripened by other methods (Figure 1C and D). These data suggest that calcium CaC_2_-ripened plantain results in alterations of plasma electrolytes when consumed.

### CaC_2_-Ripened Plantain Increased Plasma Urea and Creatinine Concentrations in Rats

**Figure 2.**
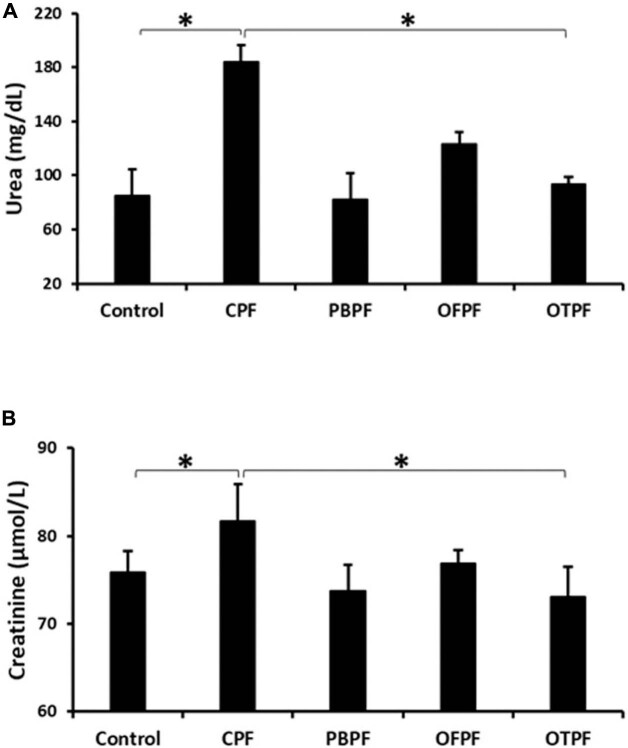
CaC_2_-ripened plantain increased plasma urea and creatinine concentration in rats. (A) Plasma urea concentration is high in rats fed with CaC_2_-ripened plantain. (B) Rats fed with carbide-ripened plantain have increased plasma creatinine concentration. CPF: Carbide-ripened plantain feed, PBPF: Polythene bag ripened plantain feed, OFPF: On-the-floor ripened plantain feed, OTPF: On-the-tree ripened plantain feed. All data are expressed as mean ± SEM (n = 3). *p < 0.05.

The effect of various methods of plantain ripening on plasma urea and creatinine concentration of rats that were fed with the plantains for four weeks is presented in Figure 2. The results indicate that CaC_2_-ripened plantain significantly increased plasma urea and creatinine concentrations when compared to rats in other groups (Figure 2A and B). These results suggests that consuming plantain ripened with CaC_2_ results in the elevation of urea and creatinine concentrations in the plasma.

### CaC_2_-Ripened Plantain Induced Glomeruli Atrophy and Tubular Necrosis

**Figure 3.**
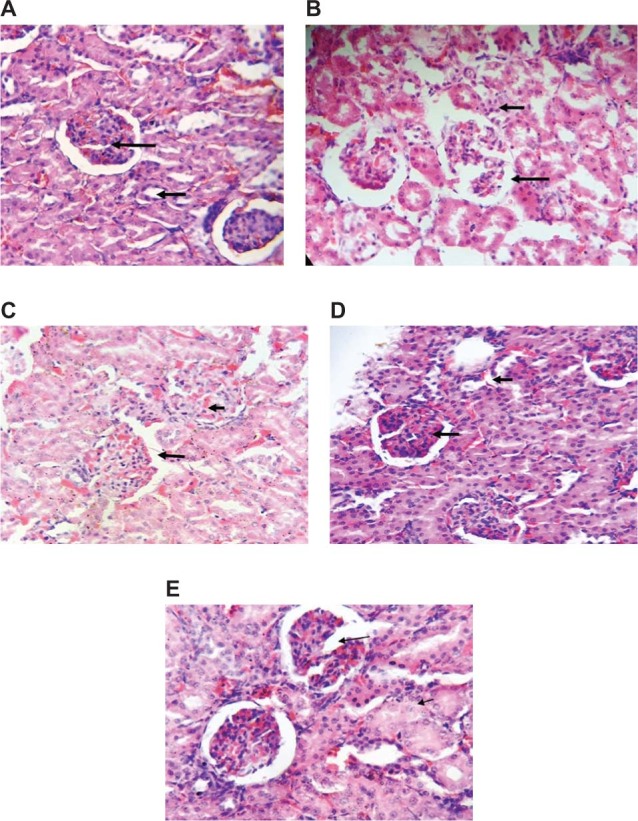
Histological analysis of kidneys from rats that received plantain-formulated diets. (A) Representative kidney from rats fed with normal animal feed. (B) Representative kidney from rats fed with CaC_2_-ripened plantain feed (CPF). (C) Representative kidney from rats fed with on-the-floor ripened plantain feed (OFPF). (D) Representative kidney from rats fed with polythene bag ripened plantain feed (PBPF). (E) Representative kidney from rats fed with on-the-tree ripened plantain feed (OTPF). n = 3.

Histological analysis was performed on the kidneys of rats that received the various diets. Kidneys from rats that received the other diets except CaC_2_-ripened plantain, showed visible renal corpuscle with glomeruli as well as normal interstitial and prominent tubules (Figure 3A, C–E). However, the kidneys of rats that were fed with CaC_2_-ripened plantain showed glomeruli atrophy and tubular necrosis. These results indicated that CaC_2_-ripened plantain was toxic to the kidneys.

## Discussion

Fruits play a vital role in human nutrition^12^, and in order to meet the increasing demand for ripe fruits, they are usually subjected to artificial ripening using various methods and chemicals such as CaC_2_ and polythene bags^23^. In this study, the physiological impact of consuming plantain ripened by artificial means was investigated.

Electrolytes are positively or negatively charged molecules called ions, which are found within the body's cells and extracellular fluids, including the plasma. The concentration of these ions is routinely analyzed to ascertain the functional status of the kidneys. The kidneys function to maintain electrolytes homeostasis, and alterations in the plasma concentrations of the electrolytes is a reflection of the functional status of the kidneys. Abnormally high concentration of sodium ion in the blood affects the osmotic pressure of the body fluid which results in an increase in the blood pressure^24^. Sodium ion is a very important extracellular electrolyte and its depletion in blood leads to dehydration and hypotension^25^. The results of this study showed that the various diets formulation did not significantly alter plasma sodium ion concentration when compared to rats in control group (Figure 1A). Our result is in agreement with the report of Igbinaduwa and Aikpitanyi-Iduitua^26^, who showed that artificially ripened plantain did not produce an alteration in plasma sodium concentration.

A significant increase in plasma bicarbonate concentration was observed in rats fed with CaC_2_-ripened plantain when compared to the other groups (Figure 1B). Bicarbonate ion plays a role in regulating the acid-base status of the blood^27^. The plasma level of bicarbonate ion is a measure of the metabolic status of the kidneys and liver^28^. Alterations in the plasma concentration of bicarbonate ion is an indication of an imbalance in the acid-base regulatory mechanism of the body^29,30^.

CaC_2_-ripened plantain feed induced a significant decrease in plasma potassium ion concentration when compared to the control (Figure 1C). Plasma concentration of potassium ions in animals fed with PBPF and OTPF were not different from those that received CaC_2_-ripened plantain feed, and the reason for this is unclear. However, this significant reduction in potassium ion concentration is an indication that artificial ripening agents or methods could result in elevated blood pressure in the consumers, since potassium ion also plays a protective role in regulating the blood pressure^31^.

Plasma concentration of chloride ion of rats fed with carbide-ripened plantain was significantly low when compared with the control and other groups (Figure 1D), and this observation agrees with the report of Igbinaduwa and Aikpitanyi-Iduitua^26^, who also reported that artificially ripened plantain produced an imbalance in plasma chloride concentration. Low plasma chloride concentration is an indication of a disturbance in acid-base balance^32^.

The progression of kidney damage is marked by the plasma level of two important chemical substances; creatinine and urea. The plasma level of urea and creatinine is used to assess glomerular filtration rate followed by renal function^33^. Creatinine is an organic base formed during muscle protein metabolism as a degradation product of creatine phosphate^34^, while urea is the major nitrogenous end product of protein and amino acid catabolism in the liver. The urea is caried in the blood to the kidneys where it's filtered by the glomerulus and excreted in the urine^35^. Therefore, the kidneys play a role in maintaining and regulating plasma urea concentration^36^. The results of this study indicate that CaC_2_-ripened plantain feed induced a significant increase in both plasma urea and creatinine concentrations when compared to the control group the other groups (Figure 2A and B). High plasma creatinine concentration is an indication of an impaired glomerular filtration which in turn results in a decreased ability of the kidneys to excrete waste products. Furthermore, histological assessment of kidneys from rats fed with carbide-ripened plantain showed glomeruli atrophy and tubular necrosis, which is an indication of a kidney damage. Our observations support the report of Ouma et al.^37^ who demonstrated a significant decrease in packed cell volume (PCV), hemoglobin, and red blood cells, as well as an increase in serum aspartate aminotransferase (AST), alanine aminotransferase (ALT), bilirubin, and pro-inflammatory cytokines such as tumor necrosis factor (TNF)-α and interferon (IFN-γ) in mice upon oral administration of CaC_2_, which indicated that CaC_2_ is toxic to body tissues. Our data, therefore, indicated a possible kidney damage or dysfunction induced by calcium carbide.

## Conclusion

The alterations observed in plasma electrolytes, urea, and creatinine concentrations may have resulted from a possible kidney damage induced by CaC_2_ used in the ripening process. Therefore, the use of chemicals such as calcium carbide in artificially ripening fruits should be discouraged because it could result in kidney damage or dysfunction in the consumers.
